# Label‐Free Quantification of Protein Density in Living Cells

**DOI:** 10.1002/cpz1.70130

**Published:** 2025-04-24

**Authors:** Robert J. Clements, Ruixin Guo, Jonathan C. Petruccelli, Michael A. Model

**Affiliations:** ^1^ Department of Biological Science Kent State University Kent Ohio; ^2^ Department of Computer Science Kent State University Kent Ohio; ^3^ Department of Physics University at Albany Albany New York

**Keywords:** cell volume, macromolecular crowding, protein concentration, refractive index, transmission‐through‐dye microscopy, transport‐of‐intensity equation

## Abstract

Intracellular water content, W, and protein concentration, P, are essential characteristics of living cells. Healthy cells maintain them within a narrow range, but often become dehydrated under severe stress; moreover, persistent loss of water (an increase in P) can lead to apoptotic death. It is very likely that protein concentration affects cellular metabolism and signaling through macromolecular crowding (MC) effects, to which P is directly related, but much remains unknown in this area. Obviously, in order to study the biological roles and regulation of MC in living cells, one needs a method to measure it. Simple and accurate measurements of P in adherent cells can be based on its relationship to refractive index. The latter can be derived from two or more (depending on the algorithm) mutually defocused brightfield images processed by the transport‐of‐intensity equation (TIE) that must be complemented by a determination of volume. Here, we describe the experimental considerations for both TIE imaging and for a particular method of cell volume measurement, transmission‐through‐dye (TTD). We also introduce an ImageJ plugin for solving TIE. TIE and TTD are fully compatible with each other as well as with fluorescence. A similar approach can be applied to subcellular organelles; however, in this case, the volume must be determined differently.© 2025 The Author(s). *Current Protocols* published by Wiley Periodicals LLC.

**Basic Protocol 1**: Sample preparation for TIE with or without TTD

**Basic Protocol 2**: Acquisition of TIE and TTD images

**Basic Protocol 3**: Calibration of TIE

**Basic Protocol 4**: Measurement of the absorption coefficient of the medium used for TTD

**Basic Protocol 5**: Image processing using Fiji

**Support Protocol 1**: Installation and use of TIE plugin

**Support Protocol 2**: Automation of the double TTD/TIE processing using a Fiji macro

## INTRODUCTION

### Significance of protein concentration and cell water content

Protein mass‐per‐volume concentration, P, and cell water content, W (the fraction of cell volume occupied by water), are basic and highly conserved characteristics of the cell; they are interrelated as (Model & Petruccelli, 2018):

(1) W≈1−0.7P.

In the rest of the text, we will be mostly discussing this dual relationship in terms of protein concentration because it is more explicitly related to macromolecular crowding (MC). The latter term refers to a condition when protein concentration is so high that it is expected to strongly affect cellular reactions due to nonspecific volume exclusion effects (Minton, 2001). These effects have been well understood theoretically and studied in vitro, but the work on living cells is only in its early stage. Presumably, one reason for the scarcity of MC studies in living cells is the difficulty of measuring it. The latter problem is addressed in this article.

Of the few established in situ results in this field, one can mention the activation of ion channels in erythrocytes by an increase in protein concentration (Colclasure & Parker, 1991, 1992), the induction of apoptosis (Rana et al., 2020), and the formation of liquid condensates (Julius et al., 2019; Subramanya & Boyd‐Shiwarski, 2024). The relationship is reciprocal, and protein concentration is in turn affected by the state of the cell. Dehydration (an increase in P) occurs in adverse conditions, such as apoptosis, senescence, starvation, sporulation, and perhaps some other types of stress (Model, in press). It has yet to be established if dehydration represents a passive response or “deliberate” adaptation serving a protective role. This is, without doubt, a fertile area for future research.

An indirect indication of the importance of protein concentration is provided by its remarkable consistency across cells of different types and origins (Model, 2021). Even when there is local water accumulation in vacuoles or necrotic blebs, the rest of the cytoplasm maintains its normal protein concentration (Hollembeak & Model, 2021; Rana et al., 2017). Moreover, cells tend to restore P following recovery from strongly hypotonic or hypertonic solutions. This process is usually formulated in terms of cell volume V: the initial and inevitable swelling in a hypotonic solution or shrinkage in a hypertonic solution are followed by ion‐driven regulatory volume decrease (RVD) or increase (RVI), respectively (Lang, 2007). As long as the cells do not grow or divide, V and P are closely related; however, in a growing cell population, cell volume is clearly not regulated while P is. Furthermore, there are cases when rapid volume regulatory responses to anisotonicity, which are usually recorded within 15 to 30 min, are absent, but slow restoration of P still occurs (Mudrak et al., 2018).

Hypotonic or hypertonic environments are by no means limited to laboratory practice. The mundane act of drinking exposes epithelial cells of the mouth and esophagus to low (or sometimes to high) osmolarity, forcing the cells to adapt; saliva is hypotonic relative to blood plasma, and urine can be either hypotonic or extremely hypertonic. Other examples of osmotic stress include the gill epithelium of fish migrating between rivers and oceans, the exposure of animals to variable salinity in estuaries, application of an antiseptic to a wound, or even opening one's eyes underwater.

### Methods for quantification of macromolecular crowding

Macromolecular crowding is created by large molecules and acts on large molecules. Some of these effects can be protein‐specific but others result from a disproportionate decrease in the volume available to all large molecules (Minton, 2001). That causes the slowing down of diffusion, a shift to more compact molecular conformations, and the assembly of monomers to oligomers.

The common methods to quantify MC are based on these anticipated effects. Measuring the diffusion rate of a fluorescent tracer is one logical approach to assess MC. However, diffusion in the cytoplasm is a complex process and can be affected by the cytoskeleton, inclusions, or metabolism‐dependent agitation of the cytoplasm; the diffusion rate of green fluorescence protein has been found to only weakly correlate with protein concentration (Cayley et al., 1991; Konopka et al., 2009).

The other group of experimental approaches utilize fluorescence resonance energy transfer (FRET) to detect conformational changes in the sensor fluorescent protein (e.g., Boersma et al., 2015; Gnutt et al., 2015; Lecinski et al., 2021; Miyagi et al., 2021). The downside of these methods is the difficulty of achieving consistent quantification that would be independent of the probe and cell type. Occasionally, FRET probes produced paradoxical results, such as an apparent increase in crowding despite an increase in cell volume (Pittas et al., 2023).

Buoyant density, which can be determined on cell populations by isopycnic centrifugation (Joyner et al., 2016) or on single cells using microfluidic techniques (Son et al., 2015) provides a more direct measure of MC. Because proteins are denser than water, the buoyant density can be converted to mass/volume concentration of proteins, with the possible limitation that the estimates of the average density of pure proteins vary within a wide range, between 1.22 g/ml and 1.43 g/ml (Fischer et al., 2004). Furthermore, the density of nucleic acids, 1.7 mg/ml (Neurohr & Amon, 2020), is significantly higher than that of proteins, and MC may be overestimated in organisms with an abundance of RNA.

The other parameter related to protein concentration is refractive index. It is linearly related to protein concentration as:

(2) $$\Delta n=n-n_{o}\approx 0.185\cdot P,$$

where n_0_ is the refractive index of the solvent (water); the refractive increment of 0.185 ml/g holds for most proteins and nucleic acids (Thiesen et al., 2020). The linearity has been observed over the concentration range of 0% to 55% (Barer & Tkaczyk, 1954). Dissolved inorganic salts have a minor effect on refractive index, and the concentration of small organic molecules (which do contribute to refractive index but produce little crowding effects) is relatively low in mammalian cells. Thus, by measuring Δn, one can, in principle, know P.

Optical coherence tomography (Khan et al., 2021) and optical diffraction tomography (Sung et al., 2009) can access refractive index directly but are complicated and expensive. Other quantitative phase imaging (QPI) methods (Mir et al., 2012) can only measure the phase delay Δϕ of light passed through the cell relative to the medium, which is related to cell thickness h as:

(3) $$\Delta \phi =\frac{2\pi h}{\lambda }\Delta n,$$

where λ is the wavelength in the vacuum. By integrating this expression over the cell area and using the relationship (2), one obtains:

(4) $$\sum \Delta \phi =\frac{2\pi V}{\lambda }\Delta n=\frac{2\pi \cdot 0.185}{\lambda }\mathrm{VP}.$$

Notice that the product VP represents the total protein mass. And since we are interested in P, the final relationship is:

(5) $$P=0.86\lambda \frac{\sum \Delta \phi }{V}.$$

It has been assumed here that protein concentration outside the cell is negligible; otherwise, P should be replaced with ΔP. Obviously, without knowledge of cell thickness, h, or cell volume, V, there is not enough information to convert phase delay into protein concentration, and protein mass will be the only available parameter.

The popular QPI method, digital holography, requires specialized illumination and optics and is therefore not readily compatible with cell volume determination. However, another QPI technique, transport‐of‐intensity equation (TIE) imaging, is perfectly suited for the task. It is based on simple brightfield (BF) transmission images that can be collected by any standard light microscope.

The nature of contrast in low‐to‐moderate resolution brightfield transmission microscopy can be understood from a simple geometric consideration. Suppose we have a cell with refractive index n immersed in a medium with refractive index n_o_. At oblique light incidence, the difference in refractive indices causes refraction of light; this makes rays converge in some areas, making them brighter, and diverge in others, making them darker. The resultant image recorded by the camera reproduces the distribution of intensity at the plane of focus; the intensity can be either real (formed by “physical” rays) or virtual (formed by continuation of “physical” rays to the focal plane). The analysis of ray propagation through a transparent sample leads to the TIE equation (Khitrin et al., 2017) for ϕ:

(6) $$\frac{2\pi n_{\mathrm{imm}}}{\lambda }\cdot \frac{\Delta I\left(x,y\right)}{I\Delta z^{\prime }}=-\nabla^{2}\phi \left(x,y\right),$$

where n_imm_ is the refractive index of the immersion medium of the objective. The parameter $\Delta z^{\prime }$ is the vertical separation between imaged planes as measured by the travel of the objective or the stage. The left‐hand side of this equation is proportional to the change in intensity observed between defocus planes. The phase ϕ on the right‐hand side is proportional to the product of cell thickness and refractive index, such that the Laplacian $\nabla^{2}$ in the transverse (x,y) coordinates characterizes the refraction of rays due to the cell's curvature and refractive index variation. This equation is slightly different from the standard TIE equation (the fact we did not point out in the original publication) in that it takes into account the presence of an external medium with refractive index n_0_ as well as the difference between the vertical shift Δz’ of the objective or the stage (the only parameter controlled by the microscope operator) and the shift of the focal plane Δz within the sample:

(7)

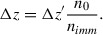




The parameter n_0_ cancels from equation (6) when both factors are accounted for. However, since solving equation (6) for ϕ requires inverting the Laplacian, which tends to amplify noise (Paganin et al., 2004; Tian et al., 2012; Zuo et al., 2017), most algorithms, including ours (Davis, 2017), incorporate denoising. In our experience, this makes the reconstructed phase an imperfect match when computed for known targets, and we find it necessary to perform calibration to replace 0.86λ in equation (5) with an empirical coefficient s:

(8) $$P=s\frac{\sum \Delta \phi }{V}.$$

For this reason, the numerical solution of equation (6) will be designated as T rather than ϕ.

The main subject of this article is the practical implementation of TIE. Numerous TIE protocols are available [e.g., Andreas, 2025; see Internet Resources], and we only describe the method based on the Fiji plugin. It has been developed at Kent State University and should be easier to use for most biologists than MATLAB‐based codes. Furthermore, since the determination of P depends on the knowledge of h or V, we describe their measurement using the same transmission microscope. The methods of cell volume measurements have been reviewed before (Model, 2018), including a publication in Current Protocols (Model, 2019); however, for the sake of completeness, the main points of the transmission‐through‐dye (TTD) method for cell volume measurement will be included here.

The TTD method is based on the exclusion of a strongly absorbing extracellular dye by cells with intact membranes. That reduces the depth of the absorbing layer, making cells appear brighter under transmitted illumination in direct relationship to their height. The resulting contrast between the intensity of a cell, I(x,y), and the background, I_bkg_, is converted into cell thickness profile, h(x,y):

(9) $$h\left(x,y\right)=\frac{\mathrm{lnI}\left(x,y\right)}{\alpha }-\frac{\ln I_{\mathrm{bkg}}}{\alpha },$$

where α is the absorption coefficient of the dye solution. To switch from TTD to TIE, one only needs to change the illumination to a wavelength where the dye absorbance is negligible.

### The content of the protocols

Basic Protocol 1 describes sample preparation when one intends to use TTD for cell volume measurements. Acquisition of TIE and TTD images on a standard brightfield microscope is the subject of Basic Protocol 2. Despite a sound theory behind TIE, the method should be calibrated, and Basic Protocol 3 describes one way to determine the coefficient s (equation 8). The conversion of TTD contrast to cell volume requires the knowledge of the absorption coefficient, α, of the contrast medium, and that is the subject of Basic Protocol 4. The computation of P from TIE and TTD images is described in Basic Protocol 5.

Support Protocol 1 explains the use of the TIE plugin for Fiji, and Support Protocol 2 describes an automated procedure for simultaneous processing of TIE and TTD images.

## Basic Protocol 1 SAMPLE PREPARATION FOR TIE WITH OR WITHOUT TTD

Any sample can in principle be used for TIE. Specifics arise from the method intended for measuring sample thickness or volume. No general recommendation can be given here; the reader will find a review of volume measurement methods in (Model, 2018). For measuring cell volumes, our preferred method is TTD. However, it is limited to whole cells with intact plasma membranes and preferably adherent to coverslips. Furthermore, cells should not be confluent to enable the determination of local background (the latter applies both to TIE and TTD).

The most common non‐TTD methods for volume measurement are fluorescence exclusion and confocal scanning. Fluorescence exclusion is like TTD, except that one measures a decrease in external fluorescence due to the presence of a cell instead of an increase in transmission (Cadart et al., 2017; Gray et al., 1983). From our experience, fluorescence exclusion is less accurate (Model, 2020) and requires microscope calibration, which is more cumbersome than determination of the absorption coefficient of the dye. Nevertheless, fluorescence exclusion has been the method of choice for some investigators. The confocal method utilizes direct labeling of the cell or the organelle. Multiple focal planes are collected, and the thickness of the sample is inferred from the depth of the fluorescent layer (reviewed in Model, 2018). This method does not depend on an intact membrane, but image processing can be more complicated. A recent application of TIE combined with a non‐TTD volume measurement can be found in (Model et al., 2024): the volume of small organelles has been estimated from brightfield images, assuming they are spherical.

### Materials

Cells
Extracellular dye with strong absorption in the visible range, such as Acid Blue 9 (AB9), also known as Brilliant Blue FCF (TCI America, cat. no. B0790), with the absorbance peak at 630 nm is required for TTD
  It can be dissolved in any cell‐compatible buffer, such as the medium in which the cells are normally kept. The optimal concentration of AB9 depends on cell height. For most cultured eukaryotic cells, 7 mg/ml provides good contrast and negligible toxicity; generally, if h’ is the distance between the coverslip and the slide necessary to accommodate the cells, the concentration of AB9 can be estimated as:
(10) $$C\left(\frac{mg}{ml}\right)\leq \frac{300}{h^{\prime }}.$$
#1.5 coverslips
Small cell culture dishes (e.g., 35 mm)
Glass slides
Lint‐free wipes
Silicon grease (can be substituted with a sticky tape of appropriate thickness or any other spacer)

*NOTE*: No reagents are needed for TIE.

1 Grow cells on loose coverslips placed in cell culture dishes. For observation, prepare a sample containing cells immersed in a solution of AB9 and sandwiched between a coverslip and a slide. To minimize artifacts in TIE images, it is essential to keep the glass clean and as free of dust as possible. Therefore, thoroughly wipe the outer surface of the coverslip with alcohol and lint‐free wipes.
  One way to prepare a sample for TTD is to put four small spots of silicon grease on the slide at distances approximately matching the length of a coverslip. The coverslip with adherent cells is picked up with forceps and placed over the grease spots with cells facing the slide. The original medium can be replaced with an AB9‐containing solution either before or after this step. The coverslip is gently pressed down until the depth of the liquid is in the right range: not too shallow, as it may crush or deform the cells, and not too deep, as this will make the sample too dark under red illumination. With some practice, the correct thickness can be easily adjusted by eye: the sample must have a sky‐blue color.
  Alternatively, strips of a thin adhesive tape can be attached to the slide. 3M Polyester Protective Tape 335 has the thickness of 40 microns and may be suitable for typical cells and a 7 mg/ml dye solution. Beads with uniform diameters added to a sample are a common technique for creating thin spacers, but we have not tried it. For an inverted microscope, neither tape nor beads will immobilize the coverslips, and they can be additionally affixed to the slide with silicone grease.
  Keep in mind that AB9 dissolved at concentration, C, increases the osmolarity, E, of solution in the range 0 to 100 mg/ml as (Model et al., 2024):
(11) $$\Delta E\left(mosm/kg\right)=2.25C\left(mg/ml\right).$$

## Basic Protocol 2 ACQUISITION OF TIE AND TTD IMAGES

This protocol describes the basic considerations for the acquisition of TIE and TTD images.

### Materials

Any optical transmission microscope with precision vertical travel of the stage or of the objective
  Two additional bandpass filters should be installed in the condenser turret or at any other convenient location between a white light source and the camera. One filter must be selective for wavelengths ∼630 nm (such as 630/10) and the other should limit light transmission to the blue range (485/10). Appropriate LED illumination can be used as well. When there is no intention to use TTD for volume measurement, any narrow‐band filter would be appropriate for TIE.
High quality grayscale camera with no less than 12‐bit resolution

1 Set up Kohler illumination (Friedman & Abramowitz, 1997) and move the 630‐nm filter into the path.
  a. The background should be dark but not black, when imaged with the camera, and the cells should be red: the thicker the cells the brighter they are.
  b. Focus on cell edges.
  c. Choose a combination of lamp brightness and exposure to make cells sufficiently bright but below saturation.
  d. Two or more images can be collected at exposures optimized for different cells within the field.
2 Switch the illumination to 485 nm. Collect two brightfield images BF1 and BF2 around the position of best focus (see Critical Parameters). The latter can be defined as a position at which the edges of cells become almost invisible (which corresponds to the plane of the coverslip for spread cells) or the contrast undergoes inversion at the edges.
  The Critical Parameters section describes the determination of the optimal step size. The quality of TIE images can be sensitive to the size of the cell‐containing area. Whenever possible, choose cells outside large clusters.

## Basic Protocol 3 CALIBRATION OF TIE

This protocol describes a method for the determination of coefficient s (equation 8).

### Materials

Silica beads of sizes comparable to the expected size of objects (e.g., from Spherotech).
  It is important to use silica because polystyrene is incompatible with some oils.
A set of oils with well‐defined refractive indices at 486 nm (n_F_) in the range 1.35 to 1.55 (Cargille Labs)

#1.5 coverslips
Glass slides
Optical transmission microscope (see Basic Protocol 2)

**Figure 1:**
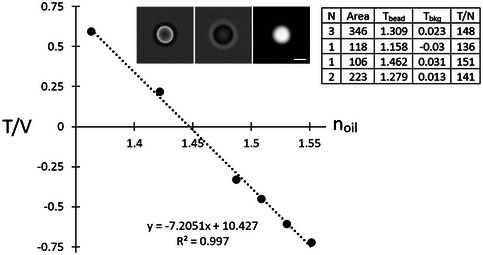
Results of calibration of a dry 20×/0.8 objective using 7.7‐µm silica beads (Spherotech, cat. no. SIP‐60‐10). The three embedded images show a bead immersed in an oil with n = 1.4218 and imaged at –2.5 µm below best focus, 2.5 µm above best focus, and the resultant TIE. The scale bar is 5 µm. The numbers in the table illustrate the calculations: the number of beads in a group (N), the selected area in µm^2^, the average T within the selected area (whose value partly depends on how the boundary was drawn), the average T of the background, and the total T per bead corrected for the background. The results for all beads are averaged and divided by the mean bead volume (239 µm^3^).

1 Thoroughly dry a small volume of a bead suspension on coverslips.
2 Overlay them with oil and mount on slides.
3 Collect a set of brightfield images of beads separated by small vertical steps. The size of the steps should be slightly less than the depth of field of the objective (Qiu et al., 2012). The beads can be either separate or in small groups. If there is a substantial camera bias (nonzero gray level in the absence of light), subtract it from all images before processing.
4 Process the images using the computational TIE solver to solve the differential equation (6) for T (see Support Protocol 1) to determine the optimal vertical step. The optimal step is characterized by sharp images, robust integral values unaffected by small deviations from the central position, and a profile best approximating a sphere.
  An example of such analysis is given in a recent publication (Model et al., 2024). We use two images taken at ± 0.5 µm (relative to the position of best focus) with an 60/1.4 oil‐immersion objective and ± 2.5 µm with a 20/0.8 dry objective.
5 Determine the average TIE reconstruction (T_bead_) for each sample of oil. To do that, draw a contour with area A around a bead or a group of beads and measure the average value of the TIE reconstruction; correct it for the value of the background:
(13) $$T_{\mathrm{bead}}=A\left(\bar{T}-T_{\mathrm{bkg}}\right).$$
  Theoretically, T_bkg_ should be zero, but the results based on actual values are preferable, especially for large areas.
  It is unnecessary to try to draw the boundary as close to the bead as possible to the bead because if a small background area is included together with the bead, it will be corrected during the operation (13).
  Beads with refractive indices substantially different from that of the oil may sometimes produce interference rings; that, however, has little effect on T_bead_.
6 Plot T_bead_ as a function of n_oil_ and construct a best fit linear regression (Fig. 1), which can be converted into:
(14) $$\frac{T_{\mathrm{bead}}}{V_{\mathrm{bead}}}=k\left(n_{\mathrm{bead}}-n_{\mathrm{oil}}\right)=g\Delta n,$$
  where the average V_bead_ is calculated from the manufacturer's data. The coefficient g is related to s in equation (8) through:
(15) $$s=\frac{1}{0.185g}.$$

## Basic Protocol 4 MEASUREMENT OF THE ABSORPTION COEFFICIENT OF THE MEDIUM USED FOR TTD

The conversion of TTD contrast into cell volume requires knowledge of the absorption coefficient, α, of the dye solution (equation 9). We prefer to measure it for every new solution (Model et al., 2008). The measurement is accomplished on an inverted microscope by a reversal of a TTD experiment: while TTD uses a dye with known α to find an object profile, the measurement of α uses an object with known profile h(x,y) to obtain α from the Beer‐Lambert law:

(16) $$\mathrm{lnI}=\ln I_{0}-\alpha h.$$

### Materials

Dye solution (see Basic Protocol 1)

Glass slides
Inverted optical transmission microscope (see Basic Protocol 2)
A half‐ball lens, 5‐mm radius (Edmund Optics)
ImageJ (https://imagej.net/ij/)
Microsoft Excel

**Figure 2:**
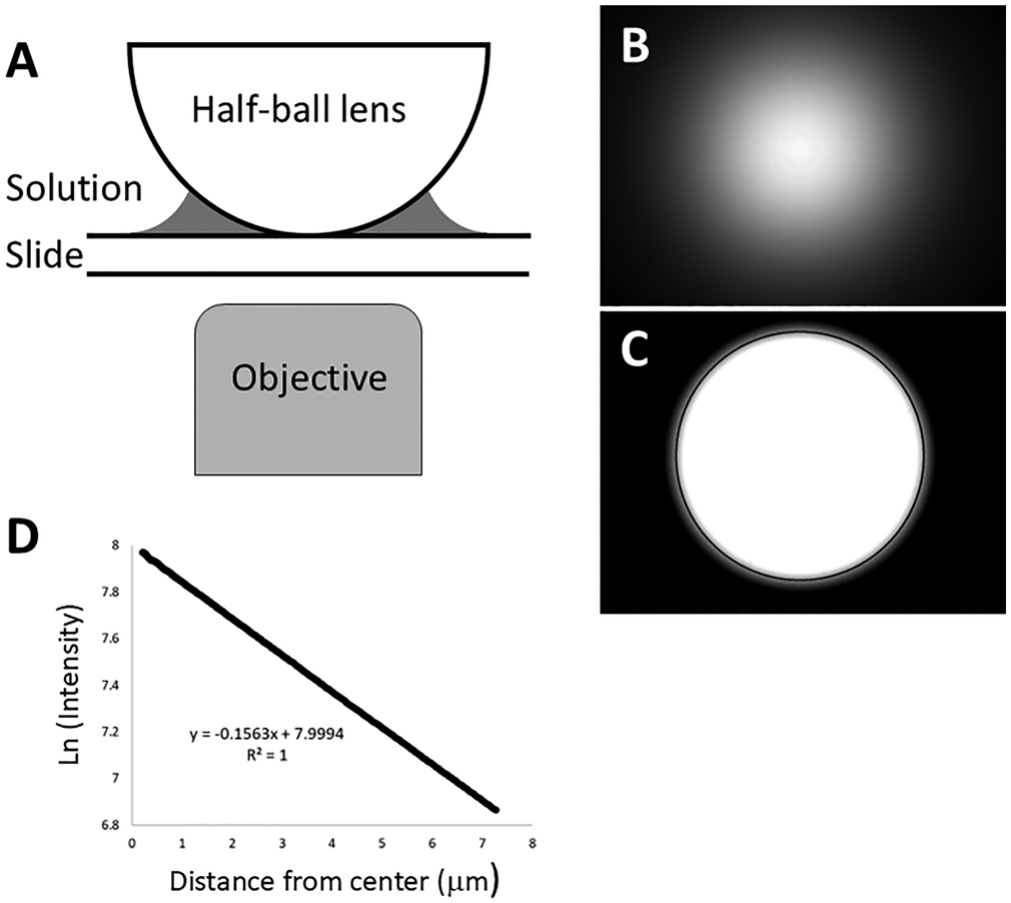
(**A**) Setup to measure the absorption coefficient of a dye‐containing solution. Both the objective and the condenser are focused on the top of the slide. A 5‐mm, half‐ball lens is immersed in a small volume of the solution; the sample is illuminated through a 635‐nm filter. (**B**) Image intensity around the touch point. The brighter center results from a smaller depth of the light‐absorbing layer. (**C**) A circle centered on the touch point is drawn on the image. Correct centering is helped by adjusting the image contrast. (**D**) Radial intensity profile is obtained by the Radial Profile ImageJ plugin; the results are exported into an appropriate software (e.g., Microsoft Excel) and fitted to a linear regression. The slope of the line gives the value of α in µm^−1^. For solutions containing 7 mg/ml AB9, α ≈ 0.15‐0.16 µm^−1^.

1 Place a small drop of solution (10 µl is sufficient) on a slide.
2 Focus an objective with sufficiently long working distance on the edge of the droplet using illumination through a 630‐nm filter (as in Basic Protocol 2).
3 Place the half‐ball lens on the slide with its curved side immersed in the solution (Fig. 2A). The point where the lens touches the slide will be bright red, and the area will grow darker with the distance from the center (Fig. 2B).

4 Focus the condenser to ensure Kohler illumination of the bottom part of the lens (since the lens distorts light propagation, this should be done after positioning the half‐ball lens).
5 Collect an image.
6 Open the image in ImageJ. Draw a circle centered on the brightest spot in the center; use appropriate contrast settings for best alignment (Fig. 2C). Apply the Radial Intensity profile (https://imagej.net/ij/plugins/radial‐profile.html) and export the intensity data to Excel.
7 Convert distances, d, to the center into depths, h, according to:
(17) $$h=5000-\sqrt{5000^{2}-d^{2}},$$
  and image intensity into logarithms (with the dark value subtracted if necessary); 5000 in equation (17) is the lens radius in µm.
  a. Plot ln(I) as a function of h, excluding the less reliable very low h values as well as the last value, which is reported by the plugin incorrectly.
  b. Fit the line to a linear regression (Fig. 2D); its slope gives the absorption coefficient.
  c. The coefficient of determination R^2^ should be no less than 0.9999.

## Basic Protocol 5 IMAGE PROCESSING USING FIJI

After the images are collected, they can be analyzed using ImageJ or Fiji (the latter is preferred for TIE). Cell volume, V, and protein mass are determined from the TTD and TIE images, respectively.

### Materials

ImageJ (https://imagej.net/ij/) or Fiji (https://imagej.net/software/fiji/downloads)
Images (from Basic Protocol 2)
Microsoft Excel

### TTD analysis

1 Set correct scale on all images (Analyze –> Set Scale…).
2 Convert TTD images into a 32‐bit floating format (Image –> Type ‐ 32‐bit).
3 Subtract the dark level if needed (Process –> Math –> Subtract…)
4 Convert the intensities into logarithms (Process –> Math ‐ Log). The symbol Log denotes the natural logarithm in ImageJ.
5 Select a region using an appropriate drawing tool. Measure the area, A, and the mean logarithmic intensity, $\bar{\mathrm{lnI}}$ (Analyze –> Measure).
6 Select an area in the background and measure its average logarithmic intensity $\ln I_{\mathrm{bkg}}$. To ensure that the background is represented correctly, you may set the display range appropriately (Image –> Adjust –> Brightness/Contrast…)
7 Copy results into Excel; compute the cell volume:
(18) $$V=\frac{A}{\alpha }\left(\bar{\mathrm{lnI}}-\ln I_{\mathrm{bkg}}\right)\;$$
  using the value of α from Basic Protocol 3.
  Naturally, if one chooses a non‐TTD method of volume measurement, the above steps are unnecessary.

### TIE analysis

**Figure 3:**
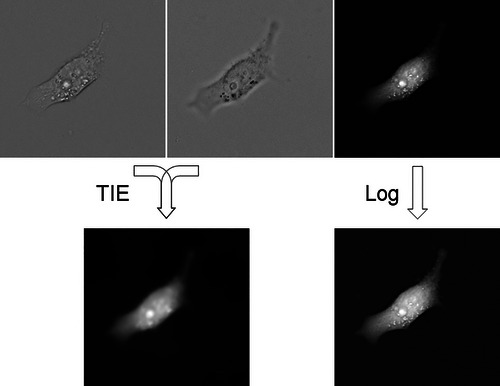
The original defocused images BF1 and BF2 and a linear TTD image are cropped to the same area. Small areas containing isolated cells or small groups of cells often produce TIE images with a more uniform background. The brightfield images are processed by TIE, and the TTD image is converted into a logarithmic scale. Note that the TIE computation removes the outer strips of pixels, so that the resultant image is 2 pixels smaller than original ones in both dimensions.

8 Arrange both brightfield images BF1 and BF2 in a stack.
9 Subtract the dark level if needed.
10 Select a rectangular region of interest; if possible, its boundaries should not cut through other cells (sometimes, that can be achieved by rotating the image). Copy the region into a separate stack (Image –> Duplicate stack).
11 Split the stack into individual images and save them.
12 Run TIE (see Support Protocol 1); measure the reconstructed values for both the cell (both the area and average) and the average background.
  TTD and TIE can be aligned before selecting the regions of interest. If the measurements are performed on matching areas (which can be automated using Support Protocol 2), there is no need to measure the area since it will be cancelled in division (equation 8).
13 Export results into Excel or a similar program. Since the coefficient s is not part of our TIE code, the correction must be introduced manually (equation 8).
  The essential steps of image processing are illustrated in Figure 3.

## Support Protocol 1 INSTALLATION AND USE OF TIE PLUGIN

To simplify calculation of TIE using Fiji, we have developed a plugin to automate image processing.

### Materials

TIE images (from Basic Protocol 2)
Fiji (https://imagej.net/software/fiji/downloads)

In order to install and utilize the software, the following steps are necessary:

1 Download Fiji and install or extract it to a folder.
2 Install the TIE plugin and dependencies using Fiji update mechanism (if this procedure does not work with your copy of Fiji download a new copy of Fiji).
  a. To accomplish this, start the Fiji updater from the toolbar: Help –> Update.

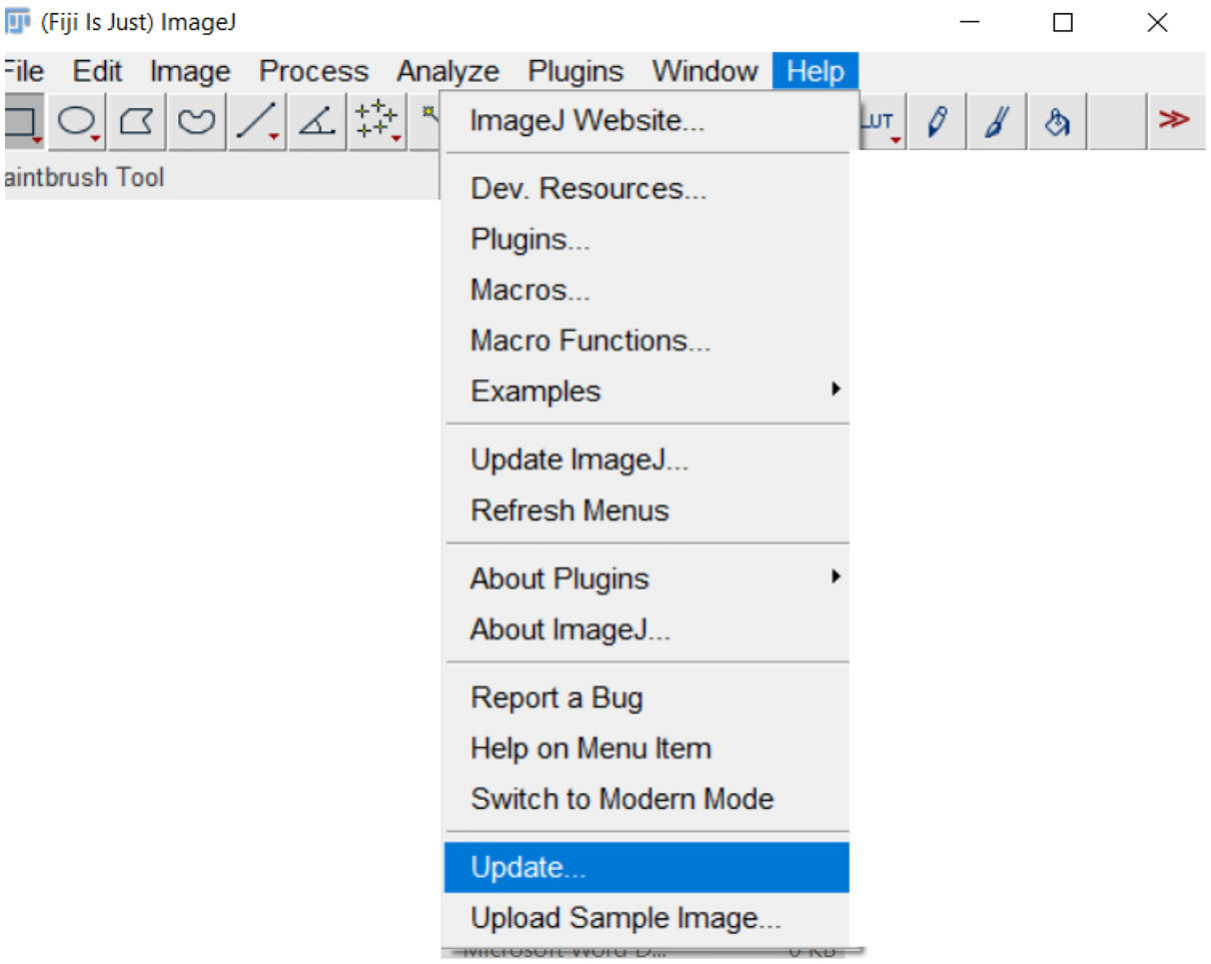

    If Fiji requests restarting, let it do so and repeat the above.
  b. Click “Manage Update Sites” from the Updater.

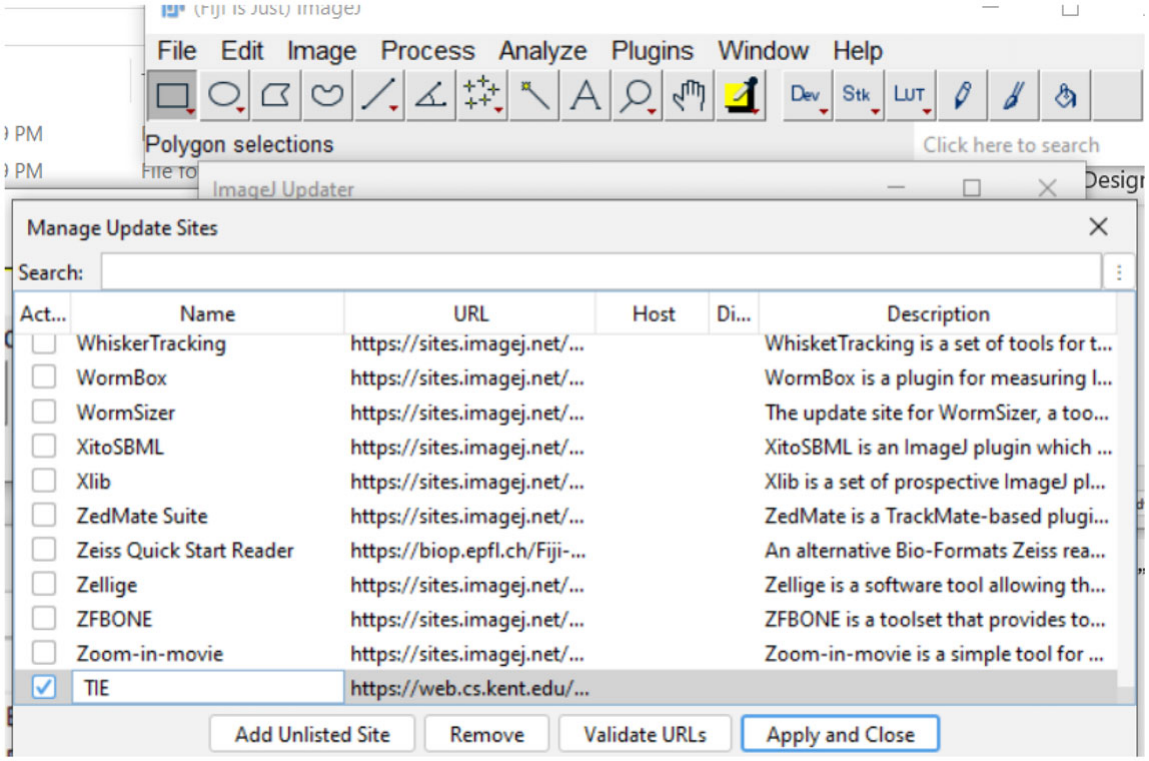

  c. Click “Add Unlisted Site” to add an additional entry.

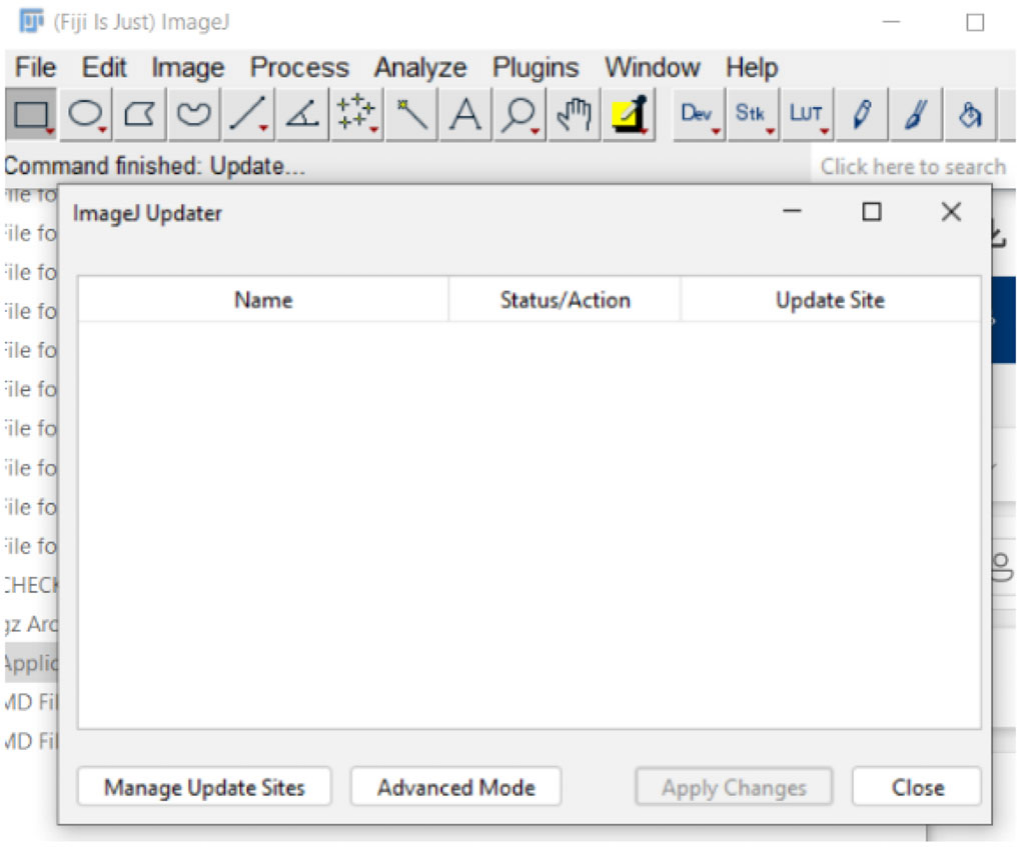

  d. Change the new unlisted sites name to “TIE” and enter the following URL: https://drosophila.biology.kent.edu/users/rclement/TIE/.
  e. Make sure the box to the left of “TIE” is checked. Click “Apply” and close, then click “Apply Changes” and restart Fiji. The TIE plugin now exists within the Fiji plugins menu as “TIEjython” and can be loaded.

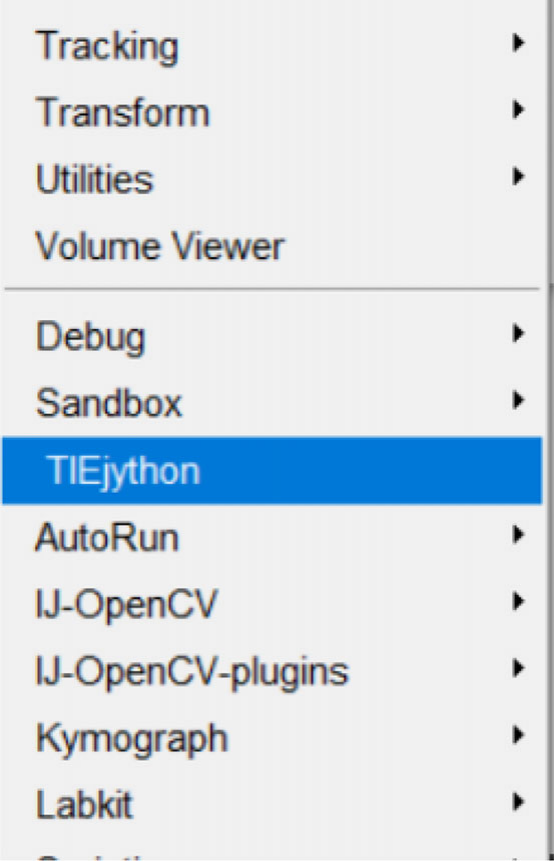

3 To use the TIE plugin, load the appropriate over‐ and under‐focused images in Fiji and run the TIE plugin.
  a. The plugin pops up a dialog box with the relevant TIE parameters.
  b. Fill in the correct experimental parameters, select the overfocused and under‐focused images, and click “OK” to perform the TIE calculation.
  c. If the “Continue Next Round” is checked, the TIEjython plugin will reopen after the calculation is performed with similar parameters populated, so the user can select another pair of images; this will continue until unchecked.

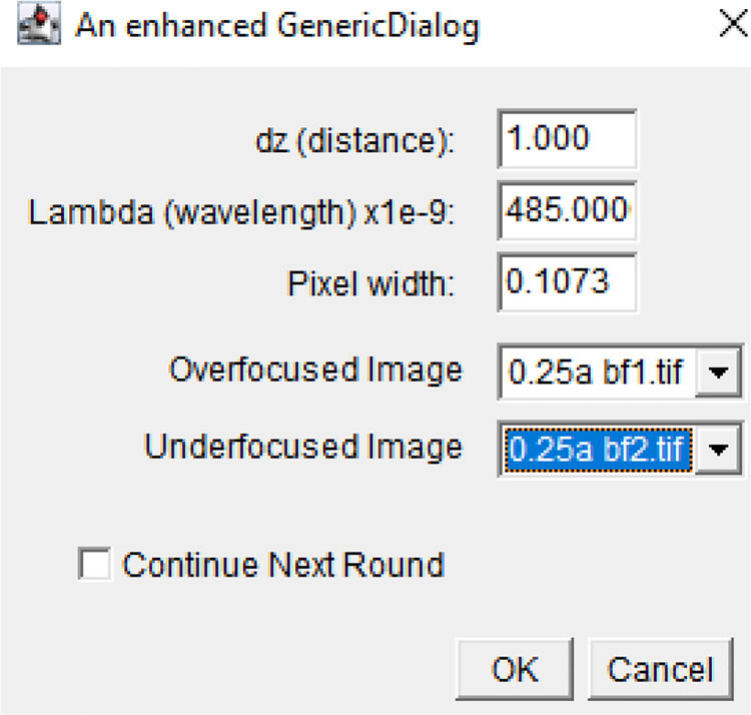

## Support Protocol 2 AUTOMATION OF THE DOUBLE TTD/TIE PROCESSING USING A FIJI MACRO

To further simplify data processing and double TTD/TIE processing, we have developed a Fiji macro that is capable of automating processing of multiple regions of interest within an image. To automate TTD/TIE processing, the developed TIEjython plugin must be installed as described in Support Protocol 1; after installation, follow the instructions below to automate TTD/TIE processing.

### Materials

TIE and TTD images (from Basic Protocol 2)
TIEautomation macro (https://drosophila.biology.kent.edu/users/rclement/TIE/TIEautomation.ijm)
Fiji (https://imagej.net/software/fiji/downloads)

1 Download the TIEautomation macro.
2 Name the files in the folder using the following scheme, so the macro recognizes the images.
  Overfocused image: [filename]bf1
  Underfocused image: [filename]bf2
  TTD image: [filename]ttd
  An example of a correctly named set of files is below:

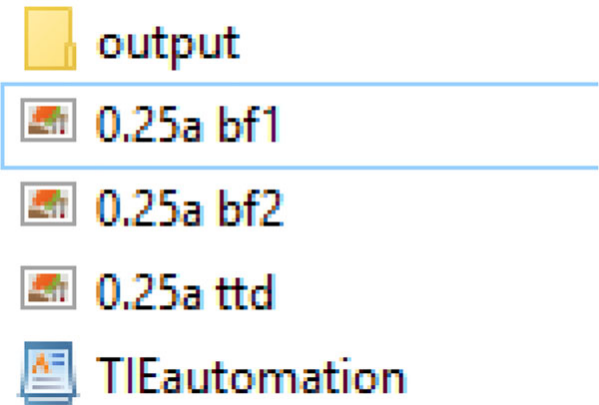

3 Open the files in Fiji or drag and drop them onto the Fiji toolbar.
4 Open the TIEautomation macro in Fiji and then click “Run”.
5 Next, the user is requested to select an output directory for saving processed images.

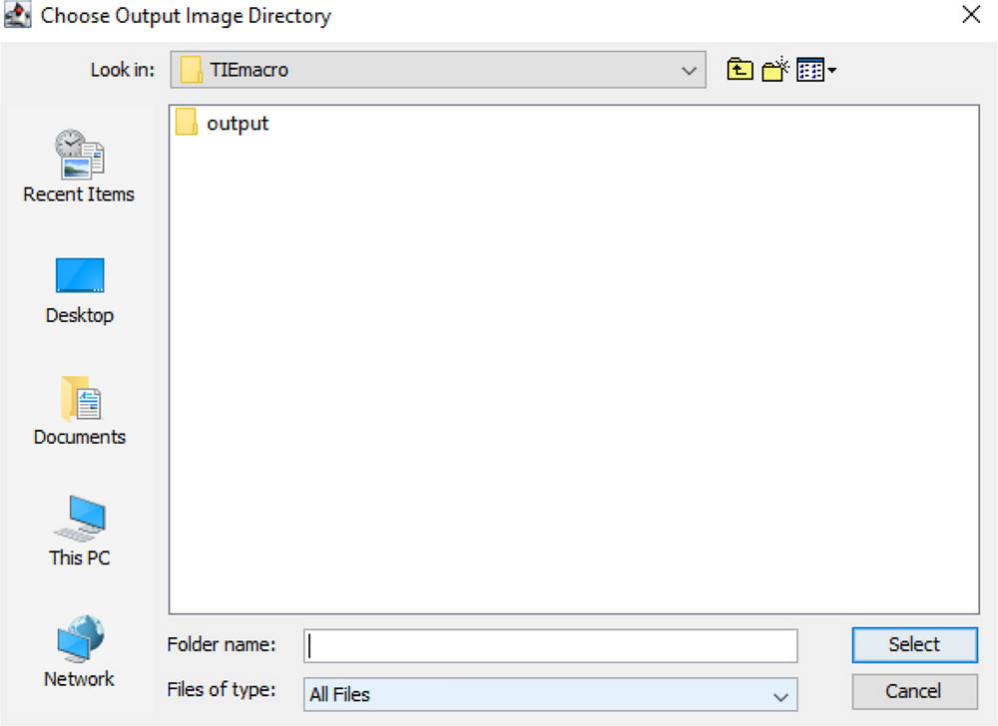

6 The macro now requests the user to enter parameters for the TIE calculation.

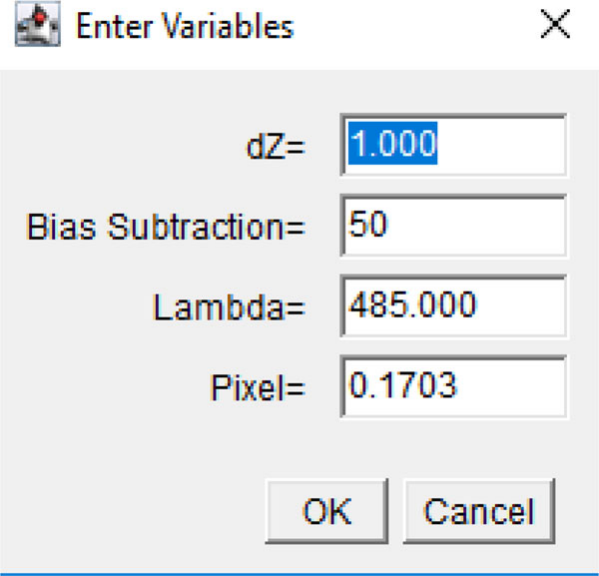

7 Next, the ROI manager is loaded, so the user can select and add ROI's in the image to analyze.

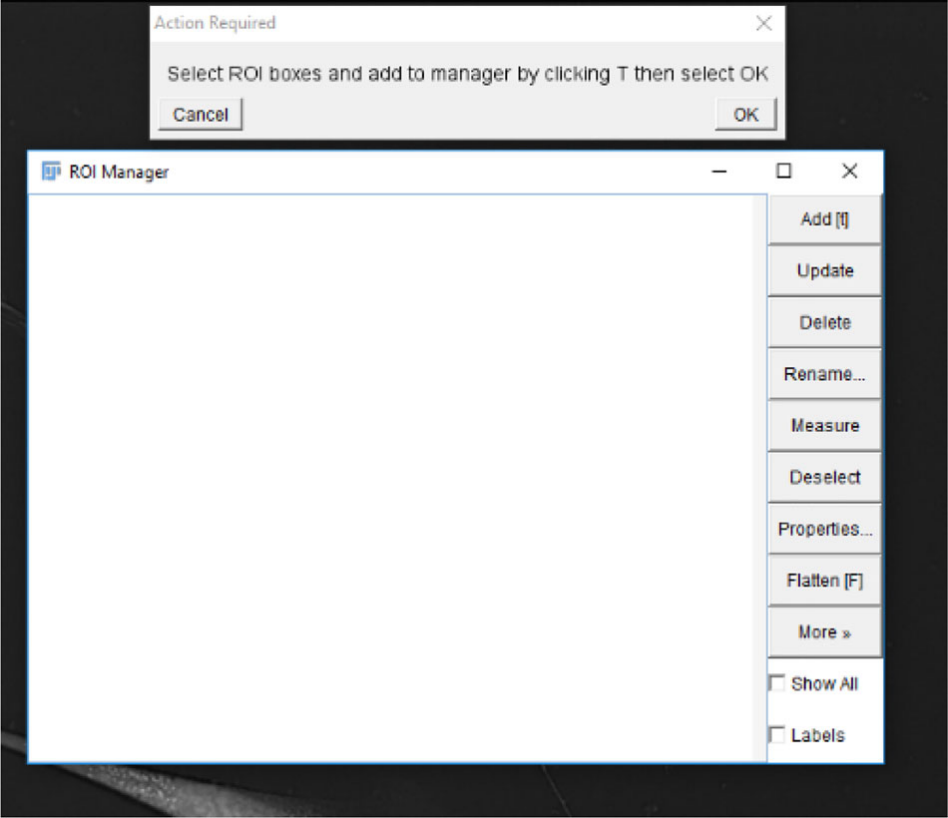

8 The user must next select the desired ROI's in the image to automatically analyze by using the bounding box (or free hand tool) to outline a cell in the image and subsequently pressing “T” to add the selection to the ROI manager.

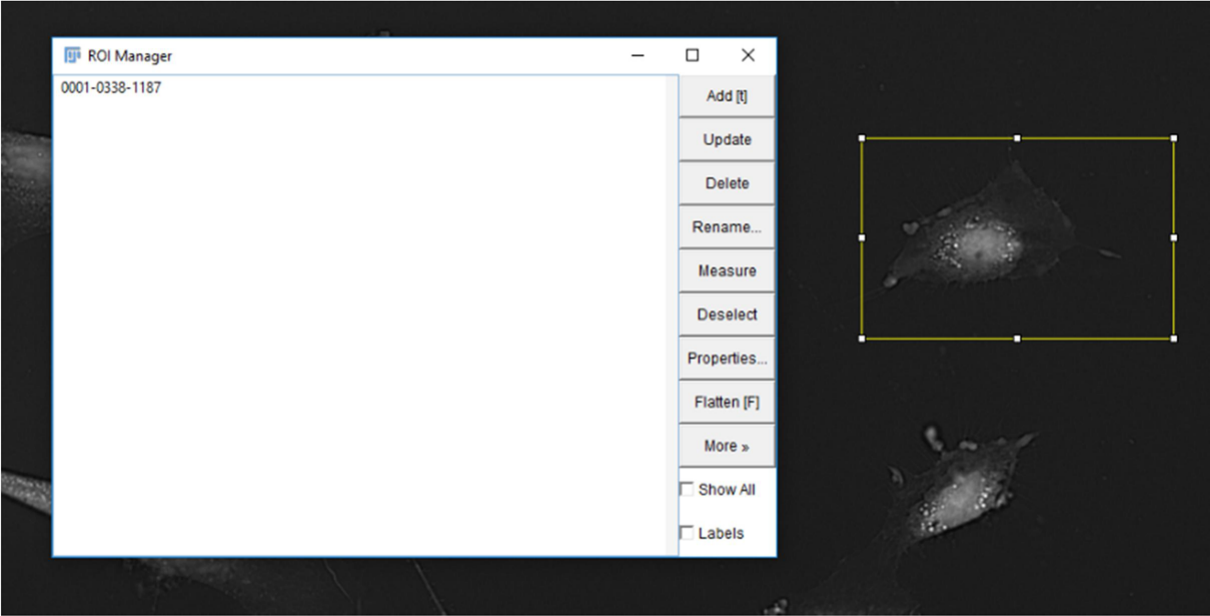

9 Repeat step 4 for all desired ROI's to evaluate in the image and then click “OK”.

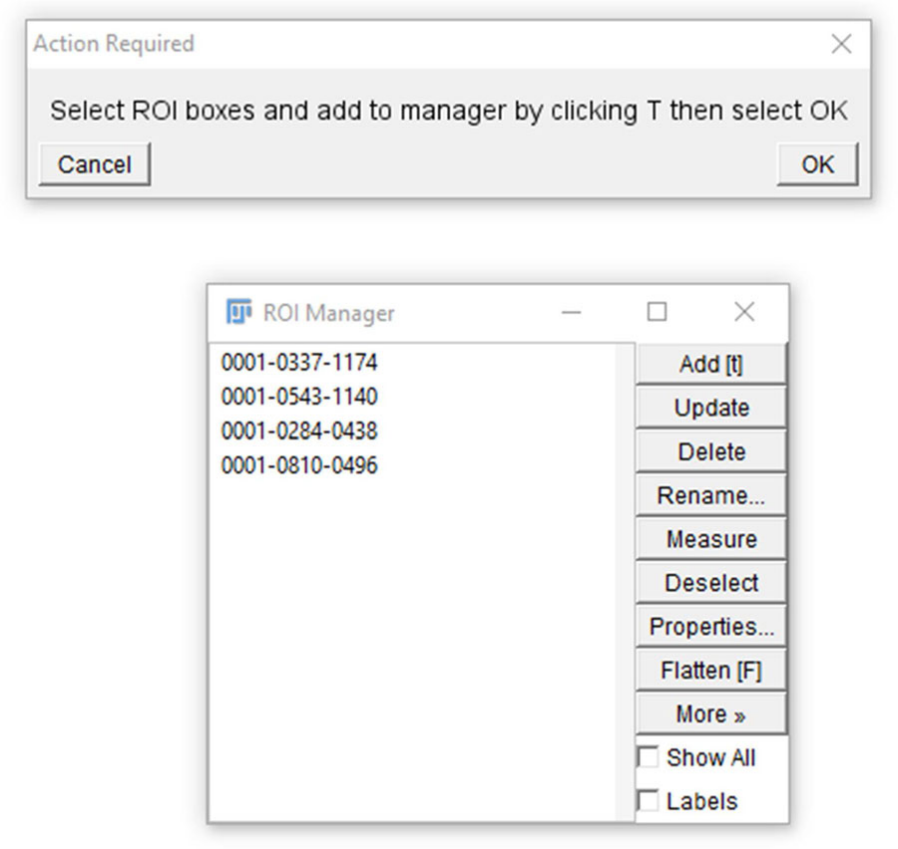

  After pressing OK do not touch the mouse or keyboard while processing is occurring.
10 Now the macro will go through all defined ROIs and perform TTD/TIE processing on each ROI sequentially, saving the output data in the previously selected folder. For each ROI the bf2 image and ttd image will be saved to the output folder, files are saved in the following format.
  TTD image: ROI_#_TTD_TIE_[filename]_ttd
  Registered Brightfield Image: ROI_#_TTD_TIE_[filename]_bf2
  As an example, files in step 1 produce the following output automatically saved to the output directory when 3 ROI's are selected.

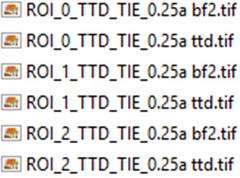

## COMMENTARY

### Critical Parameters

#### TIE

**Figure 4:**
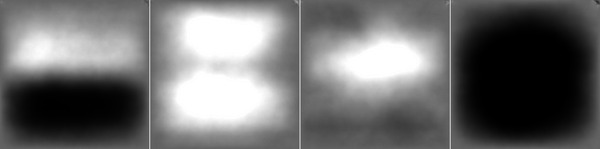
TIE images based on four pairs of blank images (transmitted illumination in the absence of a sample) collected with an ORCA‐FLASH4.0L sCMOS camera (Hamamatsu). The contrast has been digitally enhanced to display random variability. Similar results have been obtained with a cooled CCD camera (Cooke). The size of each field is 1327 µm.

**Figure 5:**
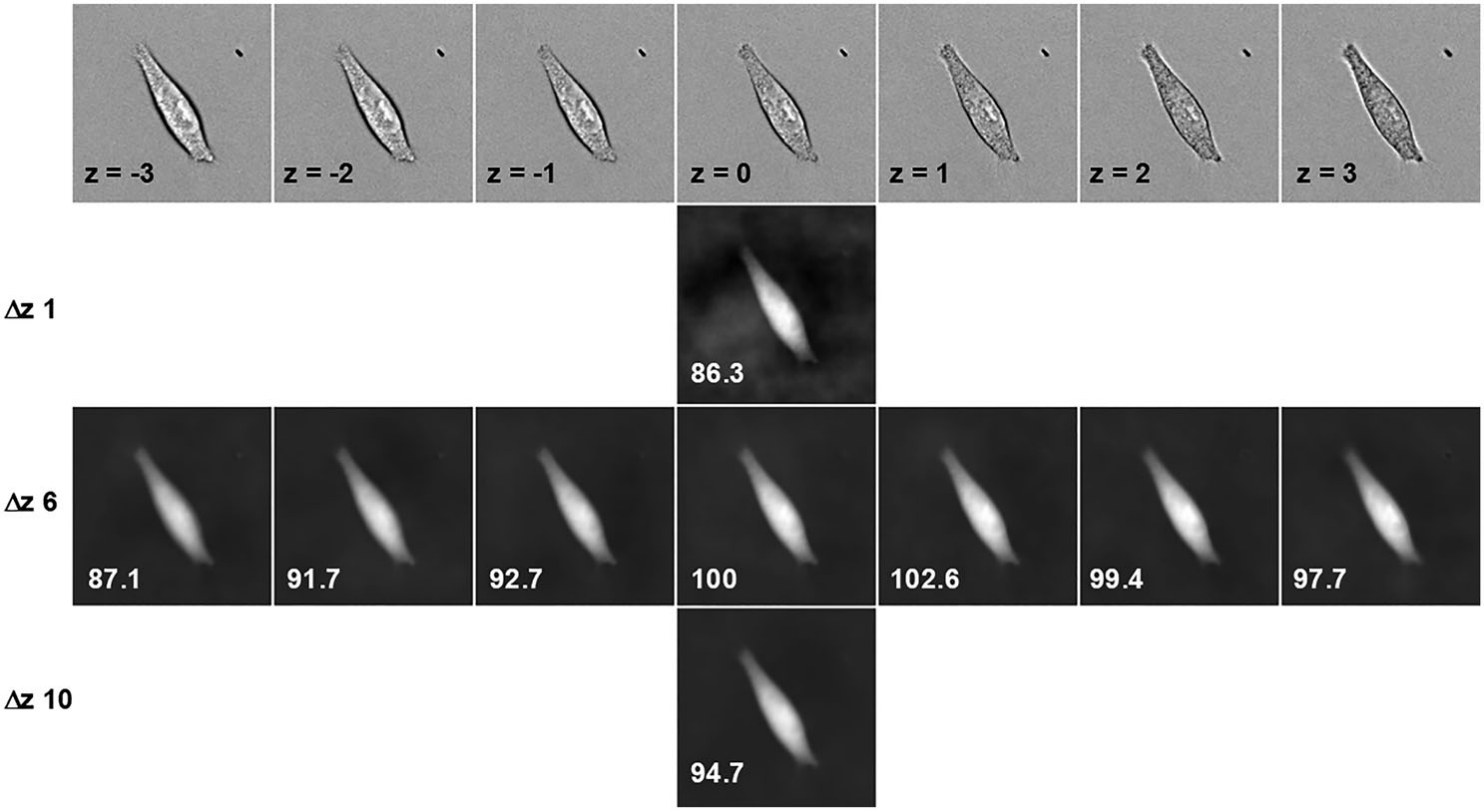
Optimization of the parameters for TIE acquisition. These images of a single HeLa cell have been obtained with a 20×/0.8 dry objective. The upper row shows brightfield images, with each successive image collected after moving the objective by 1 µm toward the cell. The image z = 0 is focused approximately on the level of the coverslip. The second, third, and fourth rows are TIE reconstructions computed at Δz separations of 1 µm, 6 µm, and 10 µm centered on the respective planes specified in the first row; for example, the second image of the third row (the one with T = 91.7 and below z = –2) has been computed from z = 1 (3 µm above z = –2) and z = –5 (not shown). The numbers in TIE images show the computed T in relative units. Small Δz result in an uneven background, and the most consistent numbers require focusing at the level of the coverslip or slightly above.

As with any type of optical microscopy, correct focusing and proper condenser adjustment are critical. Additionally, TIE depends on small differences between brightfield images BF_1_ and BF_2_; therefore, equalization of the mean intensities is included in the TIE code through the operation:

(19) $$\begin{array}{c} \mathrm{BF}1^{\prime }=\mathrm{BF}1+0.5\left(\bar{\mathrm{BF}2}-\bar{\mathrm{BF}1}\right)\\ \mathrm{BF}2^{\prime }=\mathrm{BF}2+0.5\left(\bar{\mathrm{BF}1}-\bar{\mathrm{BF}2}\right), \end{array}$$

where the bars denote average intensities. The intensities in the cell‐containing areas will of course remain different; but local unintended differences in the background may persist as well, and TIE processing will convert them into spurious darker or brighter areas. Even the residual differences by 0.0005% to 0.001% can appreciably degrade the quality of TIE. Some of them may be caused by defocused dust particles on the other side of the coverslip; but even in the absence of dust (or even in the absence of a sample, as is illustrated in Fig. 4) there are small deviations from the average that appear random and are likely related to image noise (Paganin et al., 2004; Tian et al., 2012; Zuo et al., 2017). One way to minimize their effect is to crop the cells of interest closely enough, so that the averaging operation will be more efficient throughout the image. Alternatively, one may collect not one but several images of the same plane, with a good chance that some of the images will produce better results. Also, increasing the distance between BF1 and BF2 will reduce the relative noise (albeit at the cost of sacrificing image sharpness) (Fig. 5).

When TIE is based on images defocused by a distance exceeding the depth of field, some blur is expected. If we define the best focus z = 0 as a position at which the outer edges of the cell become least visible (which coincides with the plane of the coverslip for spread cells), the most consistent results are obtained at the exact focus or at slight under‐focusing (the focal plane being above the coverslip). This is illustrated in Figure 5. When the center (located halfway between BF1 and BF2) is anywhere between z = 0 (which is visually close to best focus) and z = 3 (located 3 µm farther into the sample), T remains consistent within 2.5%. However, over‐focusing, when the center is located within the coverslip, results in a significantly lower T.

#### TTD

Theoretically, the resolvable sample thickness per pixel can be estimated as the ratio of the relative pixel noise (ΔI/I) to the absorption coefficient, α (Model et al., 2008); the latter is related to AB9 concentration as specified in equation (20). Thus, assuming ΔI/I = 0.01 and 10 mg/ml AB9, the depth 0.4 µm should be measurable. The vertical resolution will be better for features represented by many pixels; on the other hand, subtraction of the background intensity may introduce an additional error. Equation 10 provides a rule of thumb for choosing AB9 concentration based on the requirement that the background must be well above the dark level. Concentrations under 10 mg/ml are well tolerated by mammalian cells even during prolonged incubations (Gregg et al., 2010). Bacteria require much higher AB9 concentrations, such as 80 mg/ml, to obtain clear images (Lababidi et al., 2011; Model et al., 2024); however, in this case, one has to additionally consider the effect of AB9 on osmolarity:

(20) $$\mathrm{Osmolality}\left(\mathrm{mmol}/\mathrm{kg}\right)\approx 2.25C\left(\mathrm{mg}/\mathrm{ml}\right).$$

### Understanding Results

**Figure 6:**
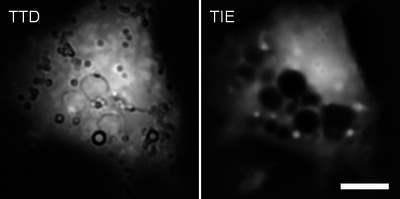
A HeLa cell with prominent vacuoles that resulted from prolonged incubation in a hypotonic medium. Since vacuoles are contained inside the cell, they produce no contrast in TTD (except from a boundary of brightfield origin). However, they reduce local protein mass, as is obvious from the TIE image. Scale bar, 10 µm.

Brighter inclusions in TIE images indicate organelles with higher protein concentration, such as nucleoli or lysosomes. Darker areas may be associated with vacuoles. In healthy cells, TIE and TTD images are often qualitatively similar, such as in Figure 3. Although intracellular inclusions visible in TIE are not supposed to generate contrast in TTD, they are often visible (Fig. 6). The reason for that is that TTD is essentially a brightfield transmission image (with an additional, and usually predominant, contribution from thickness‐related contrast), and anything visible in brightfield will also be present in TTD. Although such features can make the measurements of local thickness less reliable, they only have a minimal effect on the total volume because brighter areas are always surrounded by darker areas, and vice versa, and cancel each other in summation (Model, 2012). When vacuoles or blebs are present, they affect TIE and TTD differently. Intracellular vacuoles may not affect cell height and volume but, since they contain little protein, they show on TIE images as dark cavities. Conversely, denser organelles, such as lysosomes, generate positive contrast in TIE (Fig. 6). Thus, TIE can be applied to internal organelles, even though TTD cannot be used to quantify their volumes.

### Time Considerations

Preparation of a sample usually takes <5 min.

### Troubleshooting

A summary of common problems with TIE and TTD images is presented in Table 1.

**Table 1:** Common Artifacts in TIE and TTD

Problem	Possible cause	Solution
*TIE*		
Blurry image	BF1 or BF2 are too far from the best focal plane	Prior to collecting images, run the optimization of TIE similar to the one illustrated in Figure 5
Dark or bright “blotchy” areas in the background of TIE, as in Figure 4	Variable brightness across the field, or noise residual in BF1‐BF2 images	Crop the brightfield images to a smaller area before TIE processing
*TTD*		
The background is too dark	The distance between the coverslip and the slide is too large	Push the coverslip closer to the slide or reduce the concentration of AB9
Some cells are saturated while others are insufficiently bright	Image exposure or illumination intensity are not optimal for the existing range of cell heights	Collect two or more images optimized for different cell groups
Cells are darker than the background	Membranes are damaged	Look for other cells in the sample

### Author Contributions

**Robert Clements**: Methodology; software; supervision; validation; writing—original draft; writing—review and editing. **Ruixin Guo**: Software. **Jonathan Petruccelli**: Methodology; resources; writing—original draft; writing—review and editing. **Michael Model**: Conceptualization; investigation; methodology; project administration; resources; software; supervision; writing—original draft; writing—review and editing.

### Conflict of Interest

The authors declare no conflict of interest.

## Data Availability

Data are available from the corresponding author upon request.
